# Post-stroke sarcopenia as a neuromuscular disorder: a mini review of neural mechanisms and clinical implications

**DOI:** 10.3389/fneur.2026.1885542

**Published:** 2026-09-03

**Authors:** Lin Yang, Sen-Jun Deng

**Affiliations:** Center for Rehabilitation Medicine, Rehabilitation & Sports Medicine Research Institute of Zhejiang Province, Department of Rehabilitation Medicine, Zhejiang Provincial People's Hospital (Affiliated People's Hospital), Hangzhou Medical College, Hangzhou, Zhejiang, China

**Keywords:** corticospinal tract, neurogenic atrophy, neuromuscular junction, post-stroke sarcopenia, rehabilitation

## Abstract

**Background:**

Post-stroke sarcopenia represents a distinct neuromuscular disorder characterized by progressive muscle loss and functional decline. Unlike age-related sarcopenia, stroke-induced muscle wasting involves specific neural mechanisms originating from central nervous system injury.

**Objective:**

To systematically review the neural mechanisms underlying post-stroke sarcopenia and synthesize evidence on its clinical implications, diagnostic approaches, and therapeutic interventions.

**Methods:**

A mini review of the literature was conducted, synthesizing evidence from key studies addressing neural mechanisms, pathophysiology, diagnosis, or treatment of post-stroke sarcopenia.

**Results:**

Post-stroke sarcopenia affects 11%−67% of stroke survivors, with paretic limbs losing >30% muscle mass within 6 months. The pathophysiology involves a cascade from corticospinal tract damage to motor neuron degeneration, neuromuscular junction dysfunction, and muscle fiber atrophy. Key mechanisms include denervation-induced neurogenic atrophy, inflammatory dysregulation (IL-6, TNF-α, myostatin upregulation), and impaired IGF-1/Akt/mTOR signaling. Asymmetric muscle loss patterns distinguish this condition from primary sarcopenia. Diagnostic approaches combine imaging (DXA, BIA, CT), functional assessments (grip strength, gait speed, SPPB), and stroke-specific criteria. Multimodal interventions including neuromuscular electrical stimulation, resistance training, nutritional support, and spasticity management show promise.

**Conclusions:**

Post-stroke sarcopenia is a neurogenic disorder requiring distinct diagnostic criteria and targeted interventions. Early recognition and multidisciplinary management are essential for optimizing functional recovery and reducing disability burden.

## 1 Introduction

Stroke is still a major cause of long-term disability globally, affecting roughly 11.9 million people annually, and accounts for 10.7% of global deaths (1). Stroke survivors have progressive skeletal muscle deterioration, which severely impacts functional recovery and quality of life (2, 3). This is a distinct clinical condition, not age-related sarcopenia, because it has a different etiology, pathophysiology, and clinical presentation (4, 5).

Post-stroke sarcopenia is marked by rapid and asymmetric muscle loss, mainly affecting paretic limbs, with muscle mass reductions exceeding 30% in the first 6 months post-stroke (6). This is different from primary sarcopenia, which develops gradually via aging-related mechanisms, but post-stroke muscle wasting is caused by central nervous system injury and then the brain-muscle axis disrupted (7, 8). This neurogenic origin requires a change in how clinicians think, diagnose, and manage muscle loss in stroke survivors (9).

Even though post-stroke sarcopenia has been recognized for its clinical significance, there are many gaps in understanding its neural mechanisms and translating mechanistic insights into evidence-based interventions (10, 11). The diagnostic criteria are heterogenous, assessment time points vary, and there are limited mechanistic studies, which has made it difficult to develop stroke-specific management protocols (12, 13). Also, the upper motor neuron syndrome components, like spasticity, weakness, and altered motor control, interact complexly, which complicates the clinical picture and therapeutic approach (14, 15).

This mini review has four main objectives: (1) compile and analyze the current evidence regarding the neural mechanisms behind post-stroke sarcopenia, covering from corticospinal tract damage to muscle fiber changes; (2) evaluate the diagnostic tools and criteria that are applicable to stroke populations; (3) examine the clinical implications for functional outcomes and disability; and (4) assess therapeutic interventions targeting the neuromuscular axis. This review integrates mechanistic understanding with clinical evidence to offer a comprehensive framework for identifying and managing post-stroke sarcopenia as a unique neuromuscular disorder.

## 2 Methods

### 2.1 Search strategy and data sources

This mini-review followed PRISMA 2020 guidelines (16). We systematically searched PubMed, Embase and the Cochrane Library for articles published between January 1, 2017 and May 1, 2026, and the formal literature search was conducted on July 13, 2026. Search strategies combined terms for stroke, sarcopenia, and neural/clinical mechanisms using Boolean operators (AND, OR), MeSH/Emtree terms, and title/abstract tags. For example, the PubMed search string was: (“stroke” [MeSH Terms] OR “stroke” [Title/Abstract] OR “post-stroke” [Title/Abstract] OR “cerebrovascular accident” [Title/Abstract] OR “cerebral infarction” [MeSH Terms] OR “cerebral hemorrhage”[MeSH Terms] OR “brain ischemia” [MeSH Terms] OR “cerebrovascular disorders” [MeSH Terms]) AND (“sarcopenia” [MeSH Terms] OR “sarcopenia”[Title/Abstract] OR “muscle atrophy” [MeSH Terms] OR “muscle wasting” [Title/Abstract] OR “muscular atrophy” [Title/Abstract] OR “muscle weakness” [Title/Abstract] OR “muscle loss” [Title/Abstract] OR “skeletal muscle atrophy” [Title/Abstract] OR “cachexia” [MeSH Terms]) AND (“neuromuscular” [Title/Abstract] OR “neural” [Title/Abstract] OR “motor neuron”[Title/Abstract] OR “neuromuscular junction”[MeSH Terms] OR “denervation” [Title/Abstract] OR “reinnervation” [Title/Abstract] OR “muscle innervation” [MeSH Terms] OR “motor unit” [Title/Abstract] OR “corticospinal” [Title/Abstract] OR “neurogenic atrophy” [Title/Abstract] OR “brain” [Title/Abstract] OR “central nervous system”[MeSH Terms] OR “muscle strength” [Title/Abstract] OR “functional recovery” [Title/Abstract] OR “recovery of function” [MeSH Terms] OR “hemiplegia” [Title/Abstract] OR “paresis” [Title/Abstract] OR “grip strength” [Title/Abstract] OR “strength” [Title/Abstract]) AND (“2017/01/01”[dp]: “2026/05/01”[dp]). Equivalent strategies were adapted for Embase and Cochrane Library.

We included peer-reviewed original research, RCTs, observational studies, and reviews addressing neural mechanisms, pathophysiology, diagnosis, or treatment of post-stroke sarcopenia. Studies without mechanistic discussion, conference abstracts, animal studies lacking clinical translation, and general sarcopenia reviews were excluded.

**Figure 1:**
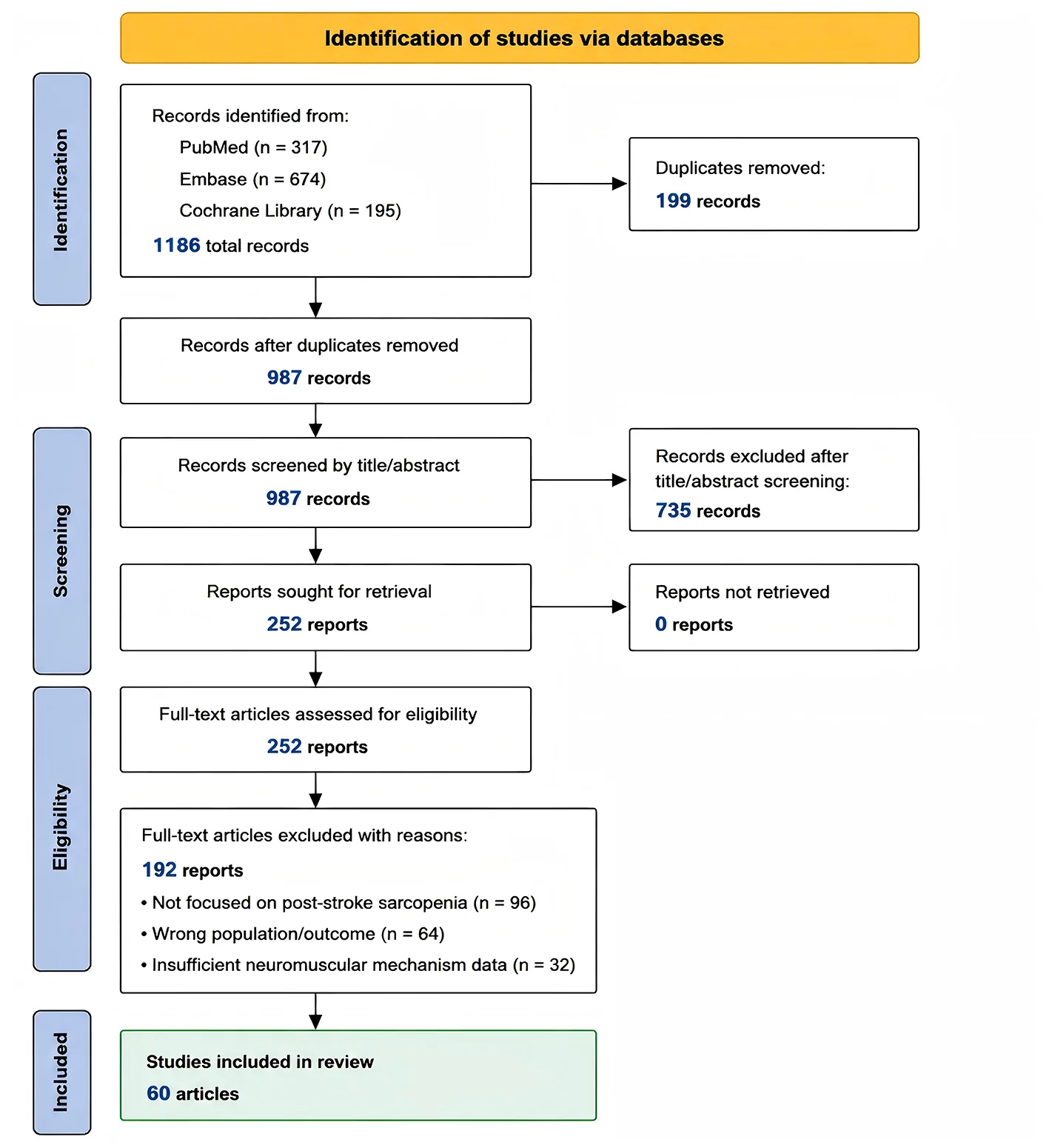
PRISMA 2020 flow diagram. From 1,186 initial records, 60 articles were included after duplicate removal, title/abstract screening, and full-text assessment. Reasons for exclusion are detailed in the diagram.

The initial search yielded 1,186 records (PubMed: 317; Embase: 674; Cochrane: 195). After removingduplicates and screening titles and abstracts, 60 articles met the inclusion criteria (Figure 1). Quality assessment was based on mechanistic clarity, evidence integration, and clinical relevance.

## 3 Results

### 3.1 Study characteristics

This mini review synthesized evidence from two complementary categories of studies. First, to elucidate the neural mechanisms of post-stroke sarcopenia (Section 3.2), we included four narrative reviews (4, 5, 7, 17), one review/commentary (6), and one original study focusing on neuromuscular changes (18). Second, to summarize clinical intervention evidence (Section 3.5), we identified six randomized controlled trials (RCTs) evaluating therapeutic strategies for post-stroke sarcopenia (Table 1). The characteristics and key findings of these RCTs are presented in Table 1.

**Table 1:** Summary of randomized controlled trials on therapeutic interventions for post-stroke sarcopenia.

References	Study design	Population (sample size)	Intervention	Control	Main outcome measures	Key findings related to post-stroke sarcopenia
Feng et al. (43)	RCT	Acute/subacute stroke survivors (*n* = 77)	Unaffected side resistance training using elastic bands, 4 weeks	Conventional rehabilitation (limb positioning, passive movements)	Handgrip strength (HG), muscle thickness (MT), FMA-UE, HAMD	URT significantly increased unaffected side HG and MT, improved upper limb function, reduced depression, and lowered sarcopenia incidence (18.4% vs 43.6% in control).
Souza et al. (49)	RCT, double-blind	Acute ischemic stroke, ≥60 years (*n* = 30)	Creatine 10g twice daily + protein (1.5g/kg/d) + early mobility, 7 days	Maltodextrin 10g twice daily + protein + early mobility	mRS, handgrip, muscle mass (BIA, ultrasound), 3-MH, inflammation biomarkers	Creatine did not improve muscle mass, strength, or function at 7d or 90d. Safe, reduced progranulin (PGRN) – a novel anti-inflammatory effect.
Nakagawa et al. (45)	RCT, open-label	Post-stroke outpatients, able to walk independently (*n* = 60)	Leucine-enriched essential amino acid jelly (10g protein, 20μg vit D) after resistance exercise, 12 weeks	Resistance exercise alone	Body composition (BIA), muscle strength (grip, knee extension), 10-m walk, 2-min walk, chair stand, FIM, renal function	Nutrition group had greater increase in BMI and less bone mineral loss vs control. No significant difference in muscle mass, strength, or function; chair stand trended better. No adverse renal effects.
Honaga et al. (47)	RCT, single-blind, placebo-controlled	Subacute post-stroke hemiparesis, rehabilitation inpatients (*n* = 50)	Whey protein 10g + vitamin D 20μg jelly twice daily, up to 16 weeks	Placebo jelly (32 kcal, no protein/vit D)	Thigh muscle CSA (CT), SMI (BIA), muscle strength, FIM-M, 10-m walk, TUG, 30-s chair stand	No significant difference in muscle mass or strength. HP group had significantly lower fat infiltration into thigh muscles (low-density CSA) and higher serum 25(OH)D, REE.
Niu et al. (11)	RCT	Acute stroke, onset 1–2 weeks, upper limb dysfunction (*n* = 77)	Upper limb end-effector robot-assisted training (4 weeks) + conventional rehab (125 min/day)	Conventional rehabilitation (140 min/day)	FMA-UE, handgrip, ASMI (BIA), muscle thickness (ultrasound), MBI	Robot group significantly improved FMA-UE, HG, ASMI, MT, MBI vs control. ASMI increased from 7.53 to 10.65 kg/m^2^ in intervention vs 7.84 to 8.62 in control.
Zhao et al. (18)	RCT, single-blind	First-ever stroke, ≤ 4 weeks, MIP < 60 cmH_2_O (*n* = 329)	Inspiratory muscle training (IMT) at 30% MIP, 30 reps × 2/day, 4 weeks + conventional neurorehabilitation	Conventional neurorehabilitation alone	Incidence of post-stroke sarcopenia (grip + calf circumference), pneumonia, MIP, mRS, MBI, TIS	IMT significantly reduced sarcopenia incidence (22% vs 36.4%), pneumonia, improved MIP, mRS, MBI, TIS vs control.

This table summarizes the six completed RCTs identified in our systematic search that evaluated exercise-based, nutritional, or combined interventions for post-stroke sarcopenia. For each trial, we extracted: (1) study design and blinding; (2) patient population and sample size; (3) intervention details (type, frequency, duration); (4) control condition; (5) primary outcome measures; and (6) key findings with statistical significance where reported. Studies were conducted in acute/subacute or chronic stroke settings, with intervention periods ranging from 7 days to 16 weeks. Abbreviations: RCT, randomized controlled trial; HG, handgrip strength; MT, muscle thickness; FMA-UE, Fugl-Meyer Assessment of Upper Extremity; HAMD, Hamilton Depression Scale; mRS, modified Rankin Scale; BIA, bioelectrical impedance analysis; 3-MH, 3-methylhistidine; PGRN, progranulin; BMI, body mass index; FIM, Functional Independence Measure; CSA, cross-sectional area; SMI, skeletal muscle mass index; CT, computed tomography; ASMI, appendicular skeletal muscle mass index; MBI, modified Barthel Index; MIP, maximal inspiratory pressure; TIS, Trunk Impairment Scale; URT, unilateral resistance training; HP, high protein; IMT, inspiratory muscle training; d, days; wk, weeks.

### 3.2 Neural mechanisms of post-stroke sarcopenia

**Figure 2:**
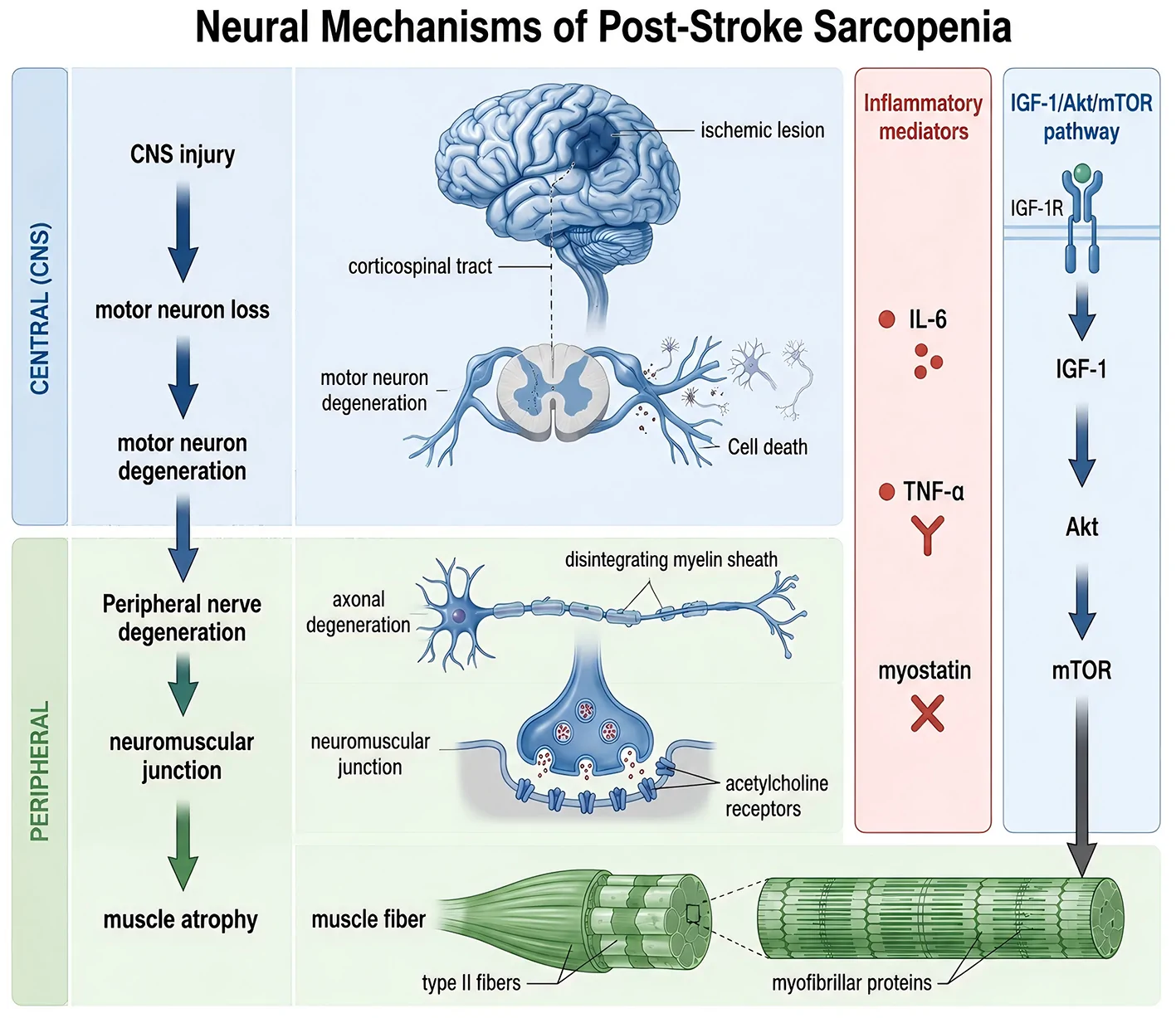
Neural Mechanisms of Post-Stroke Sarcopenia. This schematic illustrates the hierarchical pathophysiological cascade underlying post-stroke sarcopenia, progressing from central nervous system injury to peripheral neuromuscular dysfunction and muscle atrophy. The mechanisms are divided into central (upper compartment) and peripheral (lower compartment) components. Central mechanisms include ischemic brain injury, corticospinal tract disruption, motor neuron loss, and motor neuron degeneration. Peripheral mechanisms involve peripheral nerve degeneration, neuromuscular junction dysfunction with impaired acetylcholine receptor organization, preferential type II muscle fiber atrophy, and downstream alterations in muscle anabolic and catabolic signaling pathways. Molecular mechanisms include increased inflammatory mediators (IL-6 and TNF-α), elevated myostatin signaling, and impaired IGF-1/Akt/mTOR-mediated anabolic signaling. Solid arrows indicate established pathological pathways, whereas dashed arrows represent potential or hypothesized mechanisms. Color coding: blue represents neural structures and pathways; red indicates inflammatory/catabolic molecular mediators; green represents muscle structural alterations. CST, corticospinal tract; NMJ, neuromuscular junction; AChR, acetylcholine receptor; IL-6, interleukin-6; TNF-α, tumor necrosis factor-alpha; IGF-1, insulin-like growth factor 1; Akt, protein kinase B; mTOR, mammalian target of rapamycin.

The included studies consistently reveal a multi-level cascade of neural mechanisms, which connect the central nervous system injury to the peripheral muscle atrophy (Figure 2). This cascade can be thought of at four different levels: the central nervous system injury, the motor neuron degeneration, the neuromuscular junction dysfunction, and the changes in muscle fibers. Key mechanistic studies are summarized in Table 2.

**Table 2:** Summary of key mechanistic studies on neural and neuromuscular mechanisms in post-stroke sarcopenia.

References	Study design	Sample size/population	Principal mechanistic findings	Clinical relevance	Evidence level^*^
Mettler et al. (52)	Randomized controlled trial	Chronic stroke patients (*n* = 5) and healthy controls (*n* = 6)	NMES application increased phosphorylated Akt/mTOR signaling and decreased atrogin-1 expression in paretic muscle, suggesting activation of anabolic pathways and suppression of catabolic pathways.	Provides human molecular evidence that NMES may counteract neurogenic atrophy via IGF-1/Akt/mTOR pathway activation.	Human (intervention with biopsy)
Holt et al. (50)	Randomized, single-blinded crossover	Chronic stroke patients with plantar flexor weakness (*n* = 12)	A single session of chiropractic spinal adjustment significantly increased soleus V-wave amplitude (cortical drive) and maximal voluntary contraction (MVC) strength, without changing H-reflex excitability.	Demonstrates that modulating spinal afferent input can acutely enhance descending cortical drive to paretic muscles, improving motor output.	Human (neurophysiology)
Geiger et al. (51)	Randomized controlled study	Chronic stroke patients (*n* = 22)	Bi-hemispheric tDCS significantly improved maximal isometric knee extensor strength and voluntary activation, with changes correlated with baseline corticospinal excitability.	Supports the use of non-invasive brain stimulation to restore interhemispheric balance and improve neuromuscular function.	Human (neurophysiology)
Olsen et al. (53)	Randomized controlled trial	Chronic stroke patients (*n* = 20)	Endogenous paired associative stimulation (ePAS) pairing movement-related cortical potentials (MRCPs) with peripheral nerve stimulation significantly increased isometric muscle strength and voluntary activation in the paretic limb.	Provides proof-of-concept for EEG-triggered neuromodulation to enhance corticomuscular connectivity and reinnervation capacity.	Human (neurophysiology)
Navid et al. (54)	Randomized, single-blinded	Chronic stroke patients with lower limb weakness (n=29)	Chiropractic spinal adjustment increased cortical drive (V-wave) to the tibialis anterior, indicating improved supraspinal input to alpha motor neurons.	Suggests that spinal manipulation may facilitate motor recovery by enhancing descending neural drive, potentially mitigating denervation.	Human (neurophysiology)
Tanaka et al. (22)	Prospective observational	Acute stroke patients with NIHSS >3 (*n* = 51)	Quadriceps muscle thickness in the paretic limb decreased significantly within the first week. Multivariate analysis identified severe neurological deficit (higher NIHSS) and low functional mobility as independent factors for greater muscle loss.	Confirms that central neurological impairment severity is a primary driver of rapid early muscle atrophy, supporting the neurogenic model.	Human (clinical correlation)

Evidence Level: Human (neurophysiology) = direct electrophysiological measurements in stroke patients; Human (intervention with biopsy) = molecular analysis from human muscle tissue; Human (clinical correlation) = imaging/ultrasound correlated with clinical severity. Additional foundational mechanistic evidence derived from observational cohorts and narrative reviews (e.g., DTI-based tractography, MUNIX, muscle biopsy) is systematically graded in Section 4.5.

#### 3.2.1 Corticospinal tract damage and motor neuron degeneration

Stroke-induced damage to the corticospinal tract begins a cascade of downstream neural changes (6, 17). The loss of descending motor control results in trans-synaptic degeneration of spinal motor neurons, a process that is distinct from direct lower motor neuron injury (5). Studies have shown that the integrity of the corticospinal tract directly correlates with the preservation of muscle mass, with greater tract damage predicted to result in more severe muscle loss (17, 19, 20).

Motor neuron degeneration via multiple mechanisms. Cortical neurons lose trophic support, which triggers apoptotic pathways in spinal motor neurons (7, 21). Also, reduced neural activity results in decreased neurotrophic factor expression, further harming motor neuron survival (22). Electrophysiological studies show that motor unit number and activity decrease early post-stroke, which is linked to motor impairment severity (2, 3).

#### 3.2.2 Denervation and neuromuscular junction dysfunction

Motor neuron loss causes muscle fiber denervation, which is a key feature of neurogenic atrophy (4, 5, 7). Denervated muscle fibers undergo rapid atrophy, losing contractile proteins and metabolic capacity (23). The denervation process is not uniform; studies show that type II (fast-twitch) muscle fibers are more likely to be denervated than type I (slow-twitch) muscle fibers (5, 24).

Neuromuscular junction (NMJ) pathology is a key intermediate step in the neurogenic cascade (4, 25). Stroke survivors have reduced synaptic density, changed acetylcholine receptor expression, and poor neurotransmission (13, 26). Which is bilaterally, but more severely on the paretic side. Long-term denervation causes irreversible loss of neuromuscular junctions, limiting functional recovery (3, 27).

Partial reinnervation via collateral sprouting from surviving motor neurons can happen, but this compensatory mechanism is usually not enough, resulting in enlarged, unstable motor units with altered firing patterns (20, 28). The balance between denervation and reinnervation determines the final muscle loss and functional impairment.

#### 3.2.3 Inflammatory and molecular mechanisms

Post-stroke sarcopenia isn't just direct neurogenic mechanisms, it involves systemic inflammatory dysregulation (6, 7). The levels of pro-inflammatory cytokines are elevated, especially interleukin-6 (IL-6) and tumor necrosis factor-alpha (TNF-α), which cause muscle protein degradation and inhibit protein synthesis (6, 29). These cytokines activate catabolic pathways like ubiquitin-proteasome system and autophagy-lysosome pathway, speeding up muscle wasting (1, 30).

Myostatin is a negative regulator of muscle growth. In paretic muscles of stroke survivors, Myostatin is upregulated, with expression levels 40% higher than in non-paretic muscles (1). Myostatin inhibits satellite cell activation and suppresses the IGF-1/Akt/mTOR anabolic signaling pathway, further contributing to muscle atrophy (7, 31). The IGF-1/Akt/mTOR pathway is crucial for muscle protein synthesis and hypertrophy, but it's significantly impaired after stroke (7, 32). The reduced IGF-1 signaling, along with elevated myostatin and inflammatory cytokines, creates a catabolic environment that overwhelms anabolic processes (33). This molecular imbalance continues to be a factor in progressive muscle loss even in the post-acute phase (25).

#### 3.2.4 Spasticity and altered muscle architecture

Spasticity is a component of upper motor neuron syndrome, which contributes to muscle changes via multiple mechanisms, like changes in pennation angle, fiber length, and fascicle arrangement (4, 17).Chronic spasticity alters muscle architecture, including changes in pennation angle, fiber length, and fascicle arrangement (17, 34).These structural adaptations reduce muscle efficiency and force-generating capacity (35).

It's very interesting to see that the relationship between spasticity and muscle loss is quite complex. While spasticity causes immobilization and abnormal loading patterns, some studies show that spasticity might have a protective effect against muscle loss in affected limbs by keeping some muscle activation (24). This paradoxical relationship shows the multifactorial nature of post-stroke muscle changes and the need for individualized assessment.

### 3.3 Clinical manifestations and diagnostic approaches

#### 3.3.1 Prevalence and clinical presentation

Post-stroke sarcopenia prevalence varies by 11% to 67% across studies, according to diagnostic criteria, time since stroke, and population characteristics (17, 36). A recent meta-analysis found a pooled prevalence of 42% (95% CI: 33–52%) (37). In acute stroke patients, sarcopenia prevalence is 51%, which has significant effects on functional outcomes (23).

Post-stroke sarcopenia clinical presentation is asymmetric, with paretic limb having significantly more atrophy than non-paretic side (4, 6, 17). Quantitative studies show paretic limbs lose more than 30% of muscle mass in 6 months, and atrophy starts as early as 1 week post-stroke (3, 6). This asymmetric pattern is pathognomonic for neurogenic origin, and it distinguishes post-stroke sarcopenia from age-related sarcopenia (5).

Muscle loss impacts both upper and lower extremities, but the patterns vary. Lower limb muscles, especially the quadriceps, show rapid atrophy, which is strongly correlated with mobility limitations (22). Upper limb muscle loss, especially when there is shoulder subluxation, is linked to peripheral nerve conduction abnormalities and functional impairment (13).

#### 3.3.2 Diagnostic tools and criteria

Current diagnostic approaches for post-stroke sarcopenia adapt criteria from age-related sarcopenia, mainly the European Working Group on Sarcopenia in Older People 2 (EWGSOP2) and Asian Working Group for Sarcopenia (AWGS) guidelines (4, 7). But these criteria have a lot of limitations when used for stroke populations (4, 12).

##### 3.3.2.1 Muscle mass assessment

Dual-energy X-ray absorptiometry (DXA) is the gold standard for quantifying appendicular skeletal muscle mass (4, 6, 17). DXA provides accurate, reproducible measurements and can detect asymmetric muscle loss patterns (14). Bioelectrical impedance analysis (BIA) offers a more accessible alternative, though with lower precision (4, 7). Computed tomography (CT) and magnetic resonance imaging (MRI) provide detailed muscle composition analysis, including intramuscular fat infiltration, but are less practical for routine screening (6). Ultrasound is emerging as a point-of-care tool for measuring muscle thickness and quality (4).

##### 3.3.2.2 Muscle strength assessment

Grip strength measurement is a common method due to its simplicity and the ability to predict future performance (4, 6, 17). However, in stroke populations, grip strength is often confounded by hemiparesis, which requires a bilateral assessment to accurately reflect the functional capacity of the lower limb (24). Lower limb strength, assessed through quadriceps isometric testing or knee extension dynamometry, may better reflect functional capacity (14).

##### 3.3.2.3 Physical performance assessment

Gait speed, SPPB, and 6-min walk test evaluate functional capacity (4, 17). These measures correlate with disability and predict long-term outcomes (18). But stroke-related motor impairments, balance deficits, and spasticity make interpretation more complicated (12).

##### 3.3.2.4 Stroke-specific considerations

A few studies suggest that stroke-specific diagnostic criteria should account for asymmetric muscle loss, neurological impairment severity, and the time since stroke (4, 12). Proposed modifications include: (1) separate assessment of paretic and non-paretic limbs; (2) adjustment for baseline neurological function using scales like the National Institutes of Health Stroke Scale (NIHSS) or modified Rankin Scale (mRS); (3) incorporation of electrophysiological measures to assess denervation; and (4) consideration of spasticity severity (4, 24).

### 3.4 Clinical implications and functional outcomes

**Figure 3:**
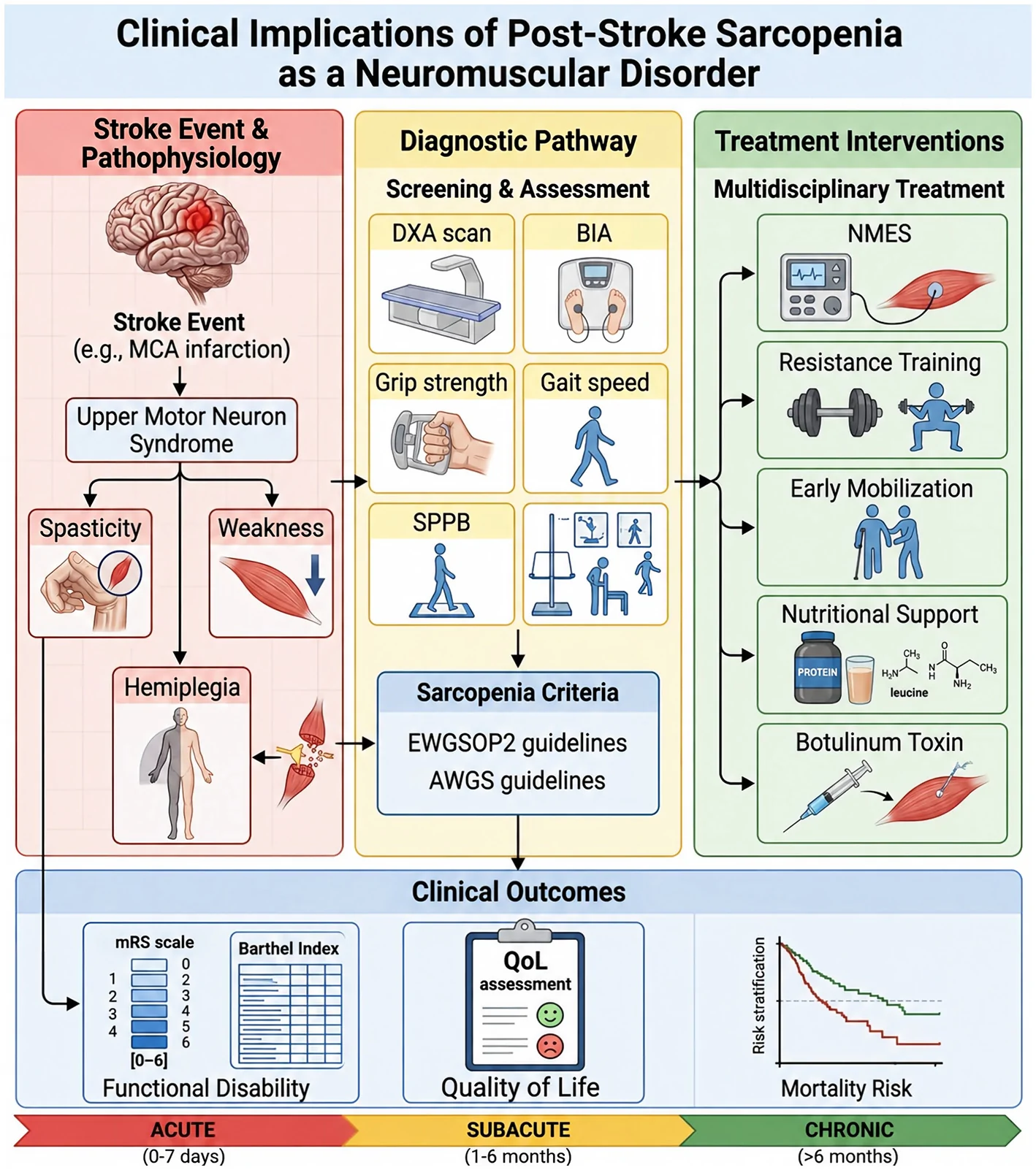
Clinical implications of post-stroke sarcopenia as a neuromuscular disorder. The clinical pathway from stroke event through diagnostic assessment to treatment interventions and outcomes. The stroke event causes upper motor neuron syndrome with spasticity and weakness, leading to hemiplegia. Diagnostic assessment includes imaging (DXA, BIA), functional tests (grip strength, gait speed, SPPB), and application of sarcopenia criteria (EWGSOP2, AWGS). Multidisciplinary treatment interventions include neuromuscular electrical stimulation (NMES), resistance training, early mobilization, nutritional support (protein, leucine), and botulinum toxin for spasticity management. Clinical outcomes encompass functional disability (mRS, Barthel Index), quality of life, and mortality risk across acute (0–7 days), subacute (1–6 months), and chronic (>6 months) phases.

Post-stroke sarcopenia has significant implications for functional recovery, the development of disability, and the long-term outcomes (Figure 3). Many studies show a strong link between muscle loss and poor functional results (6, 15, 38).

#### 3.4.1 Functional disability

Sarcopenia independently relates to poor functional outcomes at 3 months post-stroke (15). Each unit decrease in skeletal muscle mass index increases the odds of poor functional outcome (39). Conversely, improvements in muscle mass and strength correlate with better scores on the Barthel Index and Functional Independence Measure (FIM) (23, 40).

Muscle loss and disability are bidirectional. Neurological impairments limit physical activity, which can lead to disuse atrophy and muscle loss, further impairing motor function and rehabilitation potential (9). This vicious cycle necessitates early intervention to preserve muscle mass and optimize functional recovery (6).

#### 3.4.2 Cognitive function and quality of life

Emerging evidence points out that there are links between post-stroke sarcopenia and cognitive dysfunction, which might be mediated by shared inflammatory mechanisms and brain-muscle crosstalk (14). Sarcopenia is linked to worse cognitive outcomes, and may hinder rehabilitation engagement (12).

Quality of life in stroke survivors with sarcopenia is significantly reduced (28). Sarcopenia contributes to fatigue, reduced independence in daily activities, and increased caregiver burden (17, 41). Addressing sarcopenia may improve not just physical function, but also psychological well-being and social participation (42).

#### 3.4.3 Mortality and long-term prognosis

Pre-stroke sarcopenia raises the chance of poor health outcomes seven times. Post-stroke muscle loss is linked to increased mortality risk, with more muscle loss predicting worse survival (6). Sarcopenia also increases the risk of recurrent stroke, falls, fractures, and institutionalization (17, 36).

### 3.5 Therapeutic interventions

Post-stroke sarcopenia management requires a multifaceted approach, targeting both neural and muscular systems, as well as systemic mechanisms (7). Table 1 summarizes the design, intervention, comparator, and main outcomes of the six completed RCTs included in this review.

#### 3.5.1 Exercise and rehabilitation

Resistance training is the main approach to manage sarcopenia, which involves the synthesis and hypertrophy of muscle proteins. In stroke patients, progressive resistance training of both paretic and non-paretic limbs has been shown to improve muscle mass, strength, and functional capacity (43). High-intensity interval training has also been demonstrated to have potential in preventing muscle loss and modulating inflammation in experimental stroke models (4). Neuromuscular electrical stimulation (NMES) directly activates denervated or weakly innervated muscle fibers, which could potentially reduce neurogenic atrophy (44). Combing NMES with voluntary exercise enhances motor recovery and muscle preservation (1). Early mobilization is vital for preventing disuse atrophy and preserving neuromuscular function. Rehabilitation protocols that emphasize early, intensive mobilization are associated with better outcomes for muscle mass and functional recovery (6).

#### 3.5.2 Nutritional interventions

Adequate protein intake (1.2–1.5 g/kg/day) is crucial for muscle protein synthesis (7, 26). Leucine-enriched amino acid supplementation has been shown to be effective in reducing sarcopenia in post-stroke patients (45). Branched-chain amino acids (BCAAs) might improve muscle recovery by activating the mTOR pathway (46).Omega-3 fatty acids possess anti-inflammatory properties and may attenuate cytokine-mediated muscle wasting (7). Vitamin D supplementation, particularly in deficient patients, supports muscle function and may improve rehabilitation outcomes (47).

#### 3.5.3 Pharmacological and neuromodulatory approaches

Myostatin inhibitors are a promising therapeutic target, but there are limited clinical trials in stroke populations (7, 31). In hypogonadal stroke survivors, testosterone replacement may counteract the catabolic state (6).Botulinum toxin injection for spasticity management may indirectly benefit muscle health by enhancing the range of motion and allowing for more effective exercise. However, the effects on muscle mass require careful monitoring, as botulinum toxin cause localized muscle atrophy (48). Anti-inflammatory agents, like adalimumab with calcium channel modulators, have potential to improve muscle structure and function in experimental models (10). To translate this to clinical practice, further research is needed.

#### 3.5.4 Integrated care models

Optimal management demands a comprehensive, multidisciplinary approach, which includes neurologists, physiatrists, physical therapists, occupational therapists, dietitians, and nurses (14). Screening for sarcopenia should be incorporated into routine post-stroke care, with interventions tailored to individual patient characteristics, stroke severity, and rehabilitation goals (4, 12).

#### 3.5.5 Evidence quality and limitations of existing RCTs

Several limitations of the existing evidence should be noted. Five of the six RCTs enrolled fewer than 80 participants per group (11, 43, 45, 47, 49), which limited statistical power and prevented subgroup analyses. One trial did include a larger sample (*n* = 329), but its single-center design limits generalizability (18).

The intervention protocols also varied substantially across studies—covering resistance training, nutritional supplementation, robot-assisted training, and inspiratory muscle training (11, 18, 43, 45, 47, 49) making direct comparisons and protocol standardization difficult. Follow-up periods were uniformly short (7 days to 16 weeks), with no data available on long-term maintenance or hard outcomes such as recurrent stroke, falls, fractures, or mortality (11, 43, 45, 47, 49).

Outcome measures differed from trial to trial (e.g., FMA-UE, mRS, grip strength, muscle CSA, ASMI), which further complicates cross-trial comparison. Mechanistic endpoints were rarely assessed; only one trial measured biomarkers (49). Whether the observed improvements reflect neurogenic recovery, reduced inflammation, or reversal of disuse atrophy therefore remains unclear. Mechanistic work suggests that NMES may act through AMPK-ULK1-autophagy-mediated satellite cell differentiation (44), indicating that incorporating such endpoints into future trials would be informative. Finally, most RCTs were conducted in Asian populations, with no data from Western or European cohorts (11, 18, 43, 45, 47, 49).

Larger, multicenter trials with standardized protocols, consistent outcome measures, mechanistic biomarkers, and extended follow-up are needed before robust clinical guideline recommendations can be made.

## 4 Discussion

This mini review synthesizes evidence showing that post-stroke sarcopenia is a distinct neuromuscular disorder with specific neural mechanisms, clinical signs, and therapeutic benefits. The findings point out the necessity for a paradigm shift, from seeing post-stroke muscle loss a secondary effect of immobility to recognizing it as a primary neurogenic process requiring targeted intervention.

### 4.1 Post-stroke sarcopenia as a neurogenic disorder

The included studies consistently show that post-stroke sarcopenia is caused by central nervous system injury, which spreads through a hierarchical chain of motor neuron degeneration, denervation, neuromuscular junction dysfunction, and muscle fiber atrophy (4–7, 17). This neurogenic origin makes post-stroke sarcopenia different from age-related sarcopenia, which mainly involves intrinsic muscle aging processes (5, 9).The muscle loss pattern is asymmetric, with paretic limbs having significantly more atrophy than non-paretic limbs, which provides clinical evidence for the neurogenic mechanism (4, 6, 17). This asymmetry is pathognomonic and should be considered in clinical assessment and treatment planning (5).

### 4.2 Limitations of current diagnostic criteria

Current sarcopenia diagnostic criteria (EWGSOP2, AWGS) were developed for age-related sarcopenia, but they have significant limitations when used on stroke populations (4, 12). These criteria don't account for asymmetric muscle loss, neurological impairment severity, spasticity, and post-stroke changes time course (24).

A number of studies support the adoption of stroke-specific diagnostic criteria (4, 12). Proposed modifications include the assessment of paretic and non-paretic limbs separately, the adjustment for baseline neurological function, and the incorporation of electrophysiological measures to detect denervation (4, 5). The development and validation of stroke-specific criteria should be a priority for future research.

### 4.3 Toward stroke-specific diagnostic frameworks: practical recommendations and validation priorities

EWGsoP2 and AWGs criteria have known limitations in the stroke setting (4, 24). Based on the available evidence, we suggest the following clinical algorithm. Muscle mass and grip strength should be measured bilaterally; an inter-limb difference exceeding 10%−15% points toward a neurogenic etiology (4, 24). In the first 1–2 weeks post-stroke, point-of-care ultrasonography is preferable to BIA, as the latter is confounded by fluid shifts in the acute phase (4, 12). Muscle loss should be interpreted in the context of the NIHSs motor score-patients with severe paresis (score 24) require lower diagnostic thresholds and closer monitoring (24). Serial assessment at 1, 3, and 6 months is warranted; a decline in muscle thickness exceeding 5% per week should trigger immediate intervention (4).

Key priorities for validation include stroke-specific cut-off values, standardization of phase angle andecho intensity measurement protocols (12), and multi-center validation of screening tools that account for lesion location and spasticity severity (24). Neurophysiological measures such as MuNIX may help separate neurogenic atrophy from disuse atrophy (24).

### 4.4 Therapeutic implications

Post-stroke sarcopenia has a mechanistic understanding, which guides therapeutic strategies. Interventions must tackle multiple levels of the neuromuscular axis, which includes promoting motor neuron survival and reinnervation, maintaining the integrity of neuromuscular junctions, counteracting inflammatory and catabolic processes, and stimulating muscle protein synthesis (7, 8). Multimodal interventions with exercise, nutrition, and possibly pharmacological agents are the most promising (7). Early intervention is vital, since the window for motor neuron recovery and neuromuscular junction preservation is short (6). Rehabilitation protocols should prioritize muscle preservation while achieving traditional motor recovery goals (9).

### 4.5 Emerging biomarkers and neurophysiological measures

A growing set of biomarkers may improve the precision of monitoring in post-stroke sarcopenia. Among circulating markers, progranulin has drawn recent attention as an inflammatory marker responsive tointervention, alongside the more established cytokines (IL-6, TNF -a) and myostatin (6, 7, 49). on the imaging side, ultrasound echo intensity can capture intramuscular adipose and fibrous infiltration thatmay precede measurable declines in strength (4, 12, 22). Neurophysiological measures suchas MuNIx, V-wave, and H-ref lex provide direct assessments of motor neuron loss and corticospinal drive; these indices respond to neurostimulative interventions, which raises the possibility of their use assurrogate endpoints in mechanism-targeted trials (22, 24, 50). That said, most of the evidence for these biomarkers remains exploratory-sample sizes are small and protocols varyacross studies. Standardized measurement protocols and prospective validation are needed before any of these markers can be translated into clinical practice (51).

### 4.6 Gaps in evidence and future directions

Even though there are some advances in understanding post-stroke sarcopenia, there are still a lot of gaps. First, longitudinal studies with standardized assessments are needed to characterize the natural history of muscle loss, and identify critical intervention windows (12). Second, mechanistic studies using advanced imaging, electrophysiology, and molecular biomarkers can explain the relative contributions of denervation, inflammation, and metabolic dysfunction (3, 25). Third, randomized controlled trials of targeted interventions, especially those addressing neural mechanisms (e.g., neurotrophic factors, myostatin inhibitors), are lacking (7, 32). Fourth, the relationship between spasticity and muscle loss needs to be clarified, as current evidence indicates both spasticity and muscle loss have detrimental effects (17, 24). Fifth, the role of brain-muscle crosstalk and systemic factors in post-stroke sarcopenia requires further investigation, especially concerning cognitive function and the quality of life. Finally, the implementation research is needed to integrate sarcopenia screening and management into the routine stroke care pathways. Developing practical, validated screening tools and evidence-based treatment protocols will enable the translation of research findings into clinical practice (4, 12).

### 4.7 Evidence strength and knowledge gaps in the proposed neural cascade

The hierarchical model linking corticospinal injury to muscle atrophy offers a useful framework, though the strength of evidence differs across levels. Central and peripheral neural involvement is well supported in humans: DII studies correlate tract damage with the degree of muscle wasting (17, 19, 20), MuNIX confirms motor neuron loss (3, 20), and muscle biopsy reveals characteristic neurogenic atrophy patterns (5, 33). Evidence for neuromuscular junction pathology is less direct. Nerve conduction studies do show abnormalities (13, 27), but ultrastructural data come mainly from animal models (4, 7). At the molecular level, human studies consistently correlate IL-6, TNF-a, myostatin, and IGF-1 with post-stroke muscle loss (6, 29); however, the causative signaling cascades are inferred largely from rodent work (7, 30, 31).Spasticity-related architectural changes in muscle are well characterized in humans (17, 34). Takentogether, post-stroke sarcopenia can be regarded as a human neurogenic disorder, but the NMJ and intracellular signaling links require further translational study.

### 4.8 Strengths and limitations

This mini review provides a comprehensive overview of the neural mechanisms and clinical implications of post-stroke sarcopenia. Strengths include a systematic search strategy, a mechanistic focus, integration of evidence across multiple domains, and inclusion of recent high-quality reviews. Limitations include the small number of included studies (reflecting the limited literature available), reliance on narrative reviews rather than primary research, heterogeneity of populations and definitions precluding quantitative synthesis, and the possible exclusion of mechanistic insights from intervention-only RCTs.

## 5 Conclusions

Post-stroke sarcopenia is a neuromuscular disorder; it's a condition where neurogenic muscle atrophy happens because of central nervous system injury. The pathophysiology comprises a series of events, starting with the damage to the corticospinal tract, followed by the degeneration of motor neurons, the loss of denervation, the dysfunction of neuromuscular junctions, and finally, the atrophy of muscle fibers. This process is often referred to as a cascade, which involves a series of interconnected events. Asymmetric muscle loss patterns, with paretic limbs losing >30% of muscle mass within 6 months, distinguish this condition from age-related sarcopenia.

Current diagnostic criteria need to be modified to account for stroke-specific factors, like asymmetric muscle distribution, neurological impairment severity, and neurogenic origin of muscle loss. Multimodal interventions are targeting neural, muscular, and systemic mechanisms, like neuromuscular electrical stimulation, resistance training, nutritional support, and spasticity management. These interventions show promise in preserving muscle mass and optimizing functional recovery.

Post-stroke sarcopenia is a condition that requires early recognition and multidisciplinary management to reduce disability burden and improve long-term outcomes. Future research should develop stroke-specific diagnostic criteria, understand molecular mechanisms, do rigorous intervention trials, and implement evidence-based screening and treatment protocols in routine stroke care.
